# Adjoint-optimized metasurfaces for compact mode-division multiplexing

**DOI:** 10.1021/acsphotonics.1c01744

**Published:** 2022-03-07

**Authors:** Jaewon Oh, Kangmei Li, Jun Yang, Wei Ting Chen, Ming-Jun Li, Paulo Dainese, Federico Capasso

**Affiliations:** † Harvard John A. Paulson School of Engineering and Applied Sciences, Harvard University, Cambridge, Massachusetts 02138, United States; ‡ Corning Inc., Painted Post, New York 14870, United States

**Keywords:** mode division multiplexing, adjoint analysis, metasurface, space division multiplexing, fiber communication

## Abstract

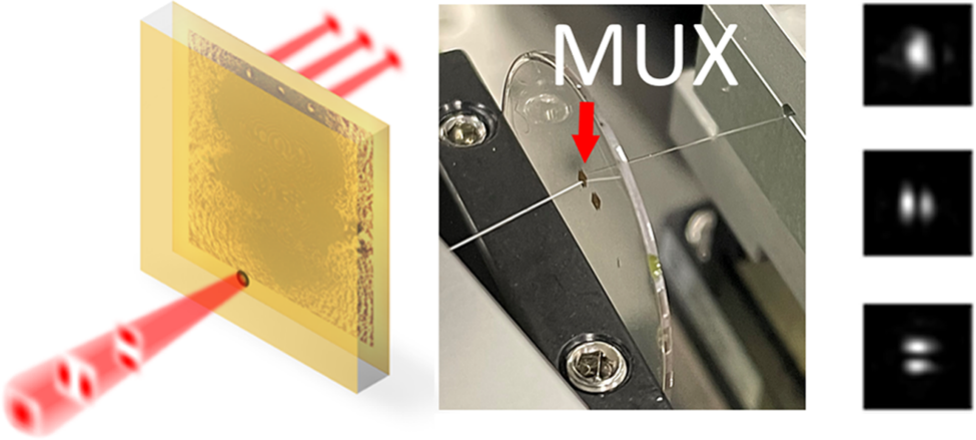

Optical fiber communications rely on multiplexing techniques that encode information onto various degrees of freedom of light to increase the transmission capacity of a fiber. However, the rising demand for larger data capacity is driving the need for a multiplexer for the spatial dimension of light. We introduce a mode-division multiplexer and demultiplexer design based on a metasurface cavity. This device performs, on a single surface, mode conversion and coupling to fibers without any additional optics. Converted modes have high fidelity due to the repeated interaction of light with the metasurface’s phase profile that was optimized using an inverse design technique known as adjoint analysis. We experimentally demonstrate a compact and highly integrated metasurface-based mode multiplexer that takes three single-mode fiber inputs and converts them into the first three linearly polarized spatial modes of a few-mode fiber with fidelities of up to 72% in the C-band (1530–1565 nm).

## Introduction

The rapid growth of data traffic due to increasing amounts of information has been driving the evolution of optical communication technology. In order to meet the demand for higher data transmission capacity, significant progress has been made to encode information in all possible dimensions of the electromagnetic field in an optical fiber: amplitude, phase, polarization, wavelength, and spatial modes.^1,2^ The spatial dimension of light is considered as the last untapped degree of freedom to further increase the capacity of communication systems. To this end, mode division multiplexing (MDM) has attracted great attention in the past decade. In an MDM system, a few-mode fiber (FMF) is used to increase the channel capacity according to the number of spatial modes that the fiber supports. The fiber mode multiplexer (MUX) and demultiplexer (DEMUX) are the key enabling devices in this system. The mode MUX excites a specific FMF mode from each input port, and the mode DEMUX is the same device operating in reverse. The main challenge in designing a mode MUX is to achieve low loss and low crosstalk as well as being scalable to many modes. A key condition to achieving low loss is to have high mode fidelity, which means that the converted mode output by the mode MUX must match the desired target mode. Broadband and polarization insensitive operation are also desired to be compatible with existing wavelength division multiplexing (WDM) systems. Finally, a compact size and ease of integration to transceivers is critical for practical implementation.

Recently, exploration into MDM has garnered great attention and, while no mode MUX/DEMUX design has been adopted for widespread use, several approaches have been reported in the literature. One method uses directional couplers that excite modes by tailoring the coupling between adjacent waveguides or fibers.^3,4^ Low loss can be achieved; however, increasing the number of accessible modes comes with the drawback of increasing device size. Fiber-based couplers also require precise control of the refractive index profile that hinders their scalability in manufacturing. Another technique uses a photonic lantern,^5^ which is composed of multiple single-mode fibers (SMFs) on one side and an FMF on the other and joined by a transition region. In this region, modes from the SMFs are adiabatically transformed into the FMF modes, providing low loss. Despite this, a photonic lantern generally suffers from high crosstalk, and its fabrication makes it difficult to scale up to higher numbers of modes. A third type of mode MUX/DEMUX is one based on free space optics.^2^ Such systems can be highly scalable;^6^ however, its implementation consists of lenses, mirrors, beam splitters, and phase plates, resulting in bulky free space setups that require precise alignment and bonding of several optical elements.^6,7^

Metasurfaces, which are defined as planar optics patterned with subwavelength-scale nanoscatterers, can often emulate and even surpass the functions of free space optics with a compact form factor. They have been shown to have control over many degrees of freedom of light including phase, amplitude, polarization, and dispersion.^8^ As a result, metasurfaces have been used to realize a multitude of different optical elements including gratings,^9^ lenses,^10−12^ and holograms,^13,14^ and they have also been used in system applications.^15,16^ In addition, their single-step lithography facilitates their adaptation to high-throughput production.^17^ Due to their subwavelength spatial resolution and low-loss dielectric composition, metasurfaces can be a suitable platform for mode multiplexing. Recently, a metasurface was used to convert the linear polarized (LP) LP_01_ mode into TE_01_ and TM_01_ modes based on Huygens’ metasurface design.^18^ However, this type of device cannot be scaled to a greater number of spatial modes and requires additional optics to couple converted modes into and out of fibers.

Here, we propose a new type of metasurface mode MUX/DEMUX that simultaneously maps from the fundamental mode to multiple spatial modes and spatially superposes the output beam for direct coupling to input/output fibers. This device is based on a metasurface cavity where light propagates back and forth between a mirror on the bottom and a reflective metasurface-based phase mask on the top surface. Because only the lateral size of the phase mask increases with additional modes, the device has potential to scale to many modes. The device is highly integrated and does not require any additional free space optics since all optical functionalities, specifically collimation, focusing, beam deflection, and mode conversion, are accomplished by the metasurface, resulting in a compact form factor that does not suffer from costly alignment like in free space systems. The metasurface is optimized by an inverse design method known as adjoint analysis,^19^ which has been used previously for shape^20^ and topology^21^ optimization of constituent nanostructures as well as cascades of optics.^22^ For our device, it provides a framework to optimize the phase profile of the metasurface in order to minimize the insertion loss. This phase profile is obtained assuming that the light propagation within the metasurface behaves as a Fabry–Perot cavity where multiple coherent reflections interfere to form the output beam. Once optimized, the phase is realized using a library of dielectric nanostructures obtained using finite-difference time domain (FDTD) simulations. As a proof of concept, we have designed and experimentally characterized a six-mode (three spatial modes and two polarization states) MUX/DEMUX that converts three SMF input channels into the first three LP modes of a FMF (LP_01_, LP_11a_, and LP_11b_) for two polarization states in the C-band. In the future, we envision this metasurface mode MUX/DEMUX as a highly-scalable platform that can control more degrees of freedom of light, including dispersion and polarization for broadband operation and manipulating vectorial modes, respectively.

## Results and Discussion

### Operating Principle of the Metasurface Mode MUX

In general, multiple optical elements are needed to shape and structure light in a desired way. Likewise, it has been shown with free space micro-optics that multiple phase plates are required to build mode MUXs with high fidelity.^6,7^ The simplest configuration would be an aligned stack of metasurfaces on separate substrates, each acting as a phase mask. The combination of multiple propagation steps and phase modulations shape the wavefront into the desired mode. However, such a design requires a highly accurate and precise alignment of the metasurface stack that impedes its scalability. Recently, there have been demonstrations of so-called “folded metasurfaces” where each phase mask is patterned on one side of the substrate and enclosed by two mirrors.^16^ This achieves a similar effect as the unfolded, aligned stack of metasurfaces, but only a single substrate is patterned and light propagation is contained within it. The fabrication is therefore much simpler since it eliminates the need for a tedious alignment process and it also avoids potential losses due to misalignment. However, by enclosing a dielectric medium with two mirrors, a cavity naturally forms. The possibility of a metasurface cavity has not been explored in previous folded devices.^16^ In such a device, light can in principle traverse the substrate indefinitely, and the output beam is the coherent superposition of all passes. The rich interaction of light with the metasurface can enable the high fidelity mode shaping required in MUX designs.

**Figure 1:**
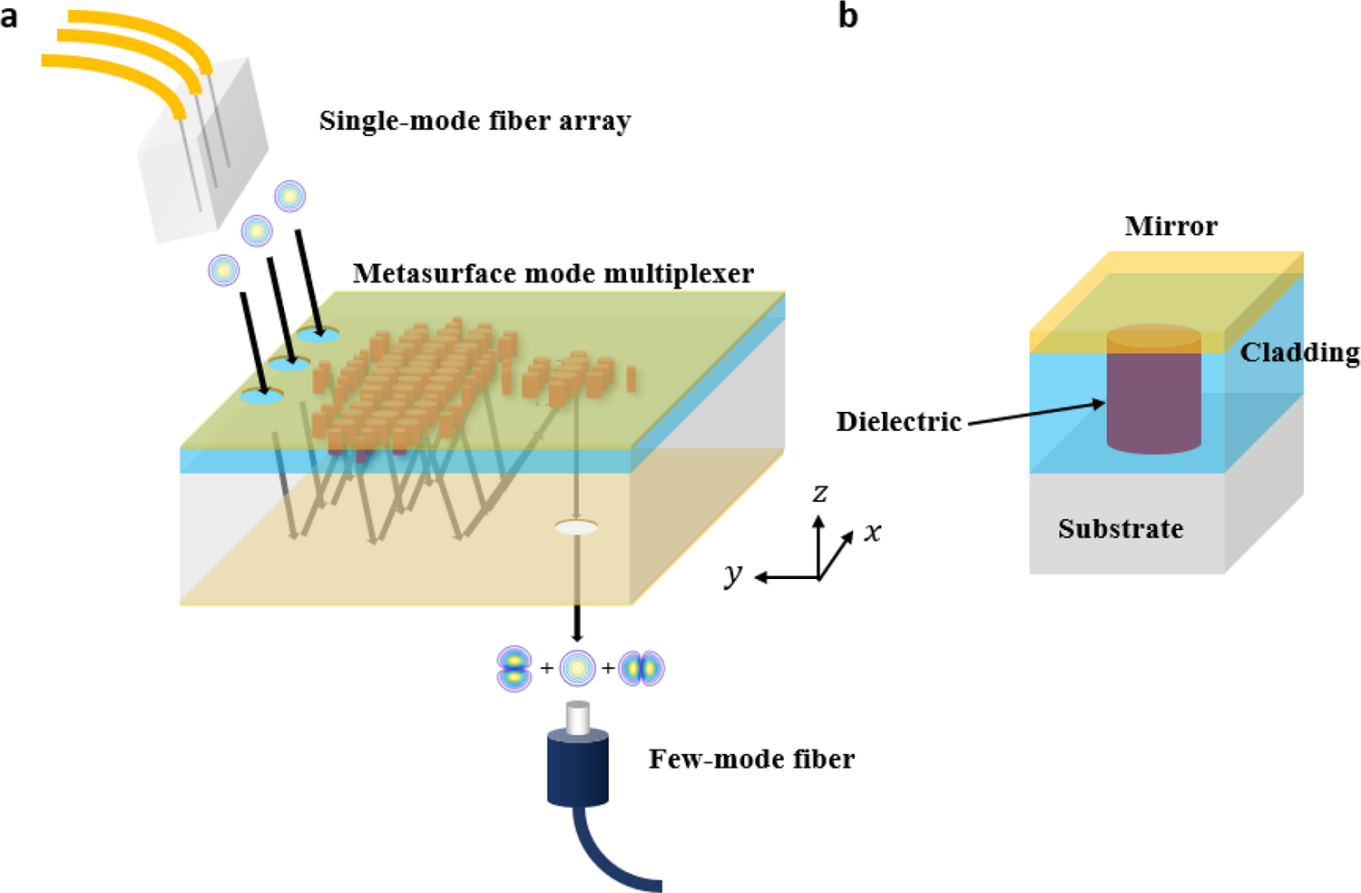
Schematic of a six-mode (three spatial modes and two polarization states) multiplexer based on a metasurface cavity. (a) Layout of the metasurface. Note the composition of the metasurface from top to bottom layer: a mirror, dielectric nanostructures encapsulated by a cladding, a substrate, and another mirror. The cladding layer exists to support the top mirror. Apertures are patterned on the top and bottom mirrors to allow light to enter and exit the device. The black arrows indicate a possible set of paths that the incident light could take to reach the output aperture. In practice, light reflects within the cavity an indefinite number of times, coherently interfering at the output. The size of the metasurface and fiber components are not to scale. (b) A unit cell of the metasurface showing an enclosed cylindrical nanostructure. In the fabricated device, the mirror is gold, the cladding is SU-8 polymer, the dielectric is amorphous silicon, and the substrate is fused silica. The bottom mirror of (a) is also gold.

Based on this cavity architecture, we design a metasurface mode MUX, and its schematic is shown in Figure 1a. Light from the facets of SMF channels enters the MUX via the input apertures patterned into the top mirror layer. Upon entering, light reflects off the bottom mirror plane and encounters the phase shift imparted by the dielectric nanopillars in Figure 1b. Then, it reflects toward the bottom mirror and back up, interacting with the nanopillars once again. This repeated interaction continuously molds the beam wavefront such that at the output, the field profile matches the target mode to be excited in the FMF positioned right below the bottom exit aperture. The same device can be used as a mode DEMUX simply by switching the input and output. In a full transmission link, a MUX couples the light into the FMF, and it is carried along the FMF until the DEMUX restores the original inputs. In Figure 1, the number of input apertures was chosen to be three to illustrate our fabricated six-mode MUX design. Nevertheless, our design can easily scale up to support more modes. Additionally, the schematic in Figure 1a shows the input and output on opposite sides of the substrate, although it is just as feasible to have both on the same side.

Note that the mode conversion must be selective to maintain low loss and low crosstalk. For example, in the context of our six-mode MUX, if only the bottom input channel is active, most input power should be converted into the LP_11b_ mode of the FMF, which is the corresponding target for that input. Similarly, if the middle (upper) input aperture is excited, only the LP_01_ (LP_11a_) mode is shown at the output. In a poorly designed mode MUX, one input channel can excite other undesired modes in the FMF, leading to high loss and high crosstalk. The fidelity of the converted mode depends on the spatial and phase resolution of the phase mask^23^ as well as the number of interactions with it. Because metasurfaces are constructed from subwavelength-sized unit cells with hundreds of phase levels, light can be shaped into a desired form even in a compact space such as within the thickness of the glass substrate. Moreover, since our device forms a cavity, the number of interactions of light with the metasurface can be large depending on the overall cavity loss.

### Adjoint Analysis of the Metasurface Phase Profile

**Figure 2:**
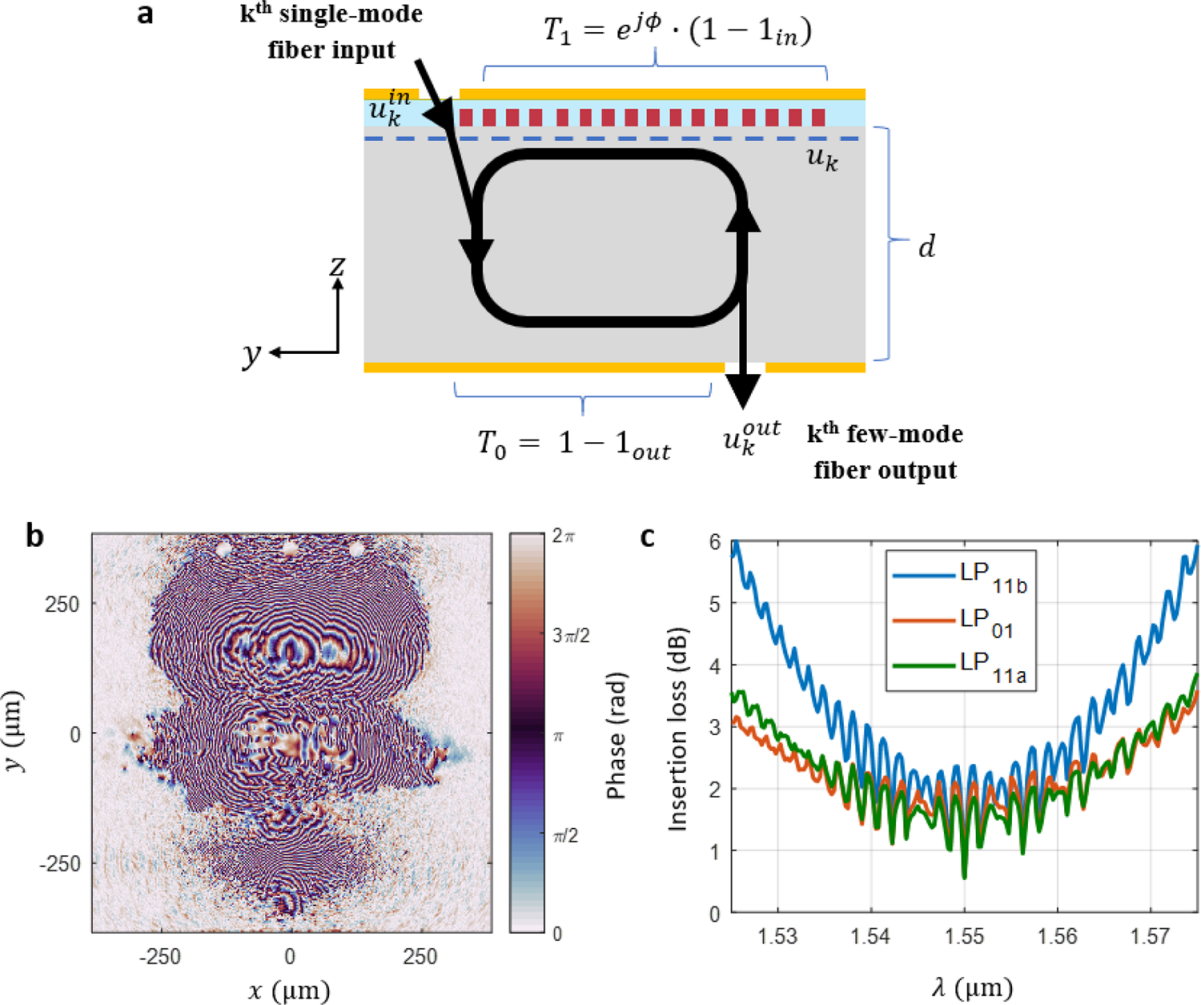
Adjoint analysis of the metasurface mode multiplexer. (a) Side view of Figure 1a with cavity model of light propagation. *u*_*k*_^in^ is the scalar field of the single-mode fiber mode that enters through the input aperture. This field propagates through the substrate of thickness *d*, reflects off the bottom reflector, propagates upward through the substrate again, is shaped by the top reflective metasurface, and then interferes with itself, ultimately reaching a stationary field *u_k_* indicated by the dashed blue line. Each time the light hits the bottom reflector, some light leaks out from the output aperture and coherently reconstructs the field into the desired few-mode fiber mode at the output aperture as *u*_*k*_^out^. (b) Optimized phase profile of a six-mode metasurface mode multiplexer and (c) its simulated insertion loss curves for all three spatial modes in the C-band. Note that the effect of the cavity model of light propagation is shown with the resonant features in the insertion loss curves.

Our metasurface device is essentially a Fabry–Perot cavity with a phase mask imprinted on one of the surfaces as illustrated in Figure 2a. For a given *k*^th^ input, we define the field from the top surface propagating downward as *u_k_*, indicated by the dashed line in Figure 2a. Under the stationary condition, *u_k_* satisfies
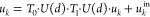
1where *u*_*k*_^in^ is the *k*^th^ input field to the device (defined at the input aperture), *d* is the thickness of the substrate, and *U*(*d*) is the free space propagator^24,25^ over distance *d* within the substrate. Due to the finite size of the device, any light that propagates outside the device region is assumed to be absorbed. *T*_1_ = 1 – 1_out_ is the transmission function for the bottom reflector, where 1_out_ is the indicator function for the output aperture, which equals 1 within the output aperture and 0 outside, ϕ is the designed phase profile on the metasurface layer, and *T*_0_ = *e*^*j*ϕ^ · (1 – 1_in_) is the transmission function for the top metasurface, where 1_in_ is the indicator function for the input aperture which equals 1 within the input aperture and 0 outside. Equation 1 is a linear equation for *u_k_* and can thus be solved efficiently using an iterative solver such as the generalized minimal residual iteration (GMRES) algorithm. Once *u_k_* is obtained, the output field is then *u*_*k*_^out^ = 1_out_ · *U*(*d*) · *u_k_*.

To help visualize *u*_*k*_^out^, one can explicitly solve *u_k_* in eq 1 by *u_k_* = (1 – *T*_0_ · *U*(*d*) · *T*_1_ · *U*(*d*))^−1^ · *u*_*k*_^in^, and with Taylor expansion , we have
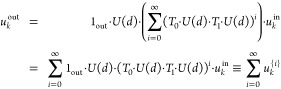
2Equation 2 can be interpreted as the following: the output field *u*_*k*_^out^ is a superposition of fields *u*_*k*_^{*i*}^, each of which originates from the input field *u*_*k*_^in^, bounces within the cavity *i* times, and then leaves the cavity through the output aperture. Furthermore, if one of the terms in the summation *u*_*k*_^{*N*}^ dominates the entire series, *u*_*k*_^out^ can be approximated by just *u*_*k*_^{*N*}^. This is then equivalent to a series of cascaded phase plates^22^ spaced equidistantly by twice the substrate thickness but enclosed back onto a single substrate. In this case, *N* is specified by design and usually scales sublinearly with the number of modes needed to be multiplexed.^7,26^ We refer to this as a “nonresonant” model as it does not include the multiple interference that could happen in the cavity.

For a mode MUX under the scalar field description, the objective function *f* can be defined as the overlap integral between the output and target mode profiles averaged over all input and output pairs
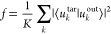
3where *K* is the number of input and output pairs. In MDM application, both the insertion loss and crosstalk are usually important. The former refers to the amount of initial launched power that is coupled into the desired output fiber channel, and the latter refers to the portion of the initial launched power that is leaked into other channels. However, if the loss is low enough, the crosstalk is low as well. Therefore, we do not explicitly include low crosstalk in our objective function. According to the modeling result that we will show later, we do automatically obtain low crosstalk because of good mode overlap.

To calculate the derivative of our objective function *f* with respect to the designed phase profile ϕ, we use adjoint analysis, which calculates the derivative  at every point (*x*, *y*) by using only two simulations
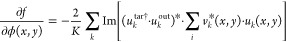
4Here, *u_k_* corresponds to the field before the metasurface in the forward propagation, and *v_k_* corresponds to adjoint field at the same place in the backward propagation. The derivation of eq 4 is given in the Supporting Information. Physically, this means that the change in the objective due to change in the phase profile is determined by the mismatch between the forward and back-propagated fields. Equation 4 is very similar to various phase retrieval techniques such as the Gerchberg–Saxton (GS) algorithm, the wavefront matching method,^27^ and multiplane light conversion.^7^ This is not surprising since these methods iteratively adjust the phase to match the fields at a target plane. However, the adjoint analysis has a more formal mathematical derivation and thus extends more naturally to complex cases such as multiple input and output pairs. In addition to optimizing the insertion loss, it is also possible to add additional objectives. For example, we added a penalty term proportional to the average phase difference between neighboring pixels. With such a term, the optimization converges toward phase profiles with smoother features, corresponding to overall higher device efficiency. Mode-dependent loss is also minimized by rephrasing *f* into a min–max problem, that is, to minimize the worst-case loss among all input modes. Given the derivatives of the objective function, a gradient-based optimization method, such as the Broyden–Fletcher–Goldfarb–Shannon (BFGS) algorithm, is used to optimize design parameters until the performance converges.

In practice, due to the high cost in solving a large linear equation (eq 1) and the intrinsic sensitivity of design parameters in a resonant structure, which leads to slow convergence, we initialize the design parameters using the optimized phase profile for a nonresonant model. We pick *N* = 3, which we find to be a good compromise between high fidelity in mode conversion and low number of interactions for high efficiency. Then the overall design flow is the following: (1) optimize the phase profile using adjoint analysis, assuming a nonresonant model; (2) use the optimized phase profile as the starting point in the second optimization, assuming a resonant model. The second optimization gives the final optimized phase profile of the device.

Shown below are the results of the adjoint method optimization of the six-mode MUX (consult the Methods section for a detailed description of the parameters used to simulate this device). Table 1 contains the optimized insertion loss and crosstalk (in dB) of the device at the design wavelength. Figure 2b shows the optimized phase profile of the metasurface MUX. Note the three regions in the phase that correspond to the initial condition where we assumed three reflections within the cavity. Although the cavity model assumes a steady-state field where light is reflecting indefinitely within the substrate, the remnants of the initial phase are apparent. The simulated insertion loss of the design is ∼0.6 dB at 1550 nm for all three modes (Table 1). This includes two contributions: mode fidelity (mismatch between output beam shape and target mode shape) as well as any diffraction loss (i.e., light that gets scattered outside the device and never exits through the output aperture). The modeled crosstalk between any two channels is below −30 dB. The insertion loss of each mode over the C-band is plotted in Figure 2c. The curve has periodic peaks and valleys indicative of the free spectral range of a resonant cavity. Note also that the mode-dependent loss was minimized (<0.05 dB) as all three modes converge to a similar insertion loss at 1550 nm.

**Table 1:** Simulated Insertion Loss and Crosstalk (dB) at 1550 nm of the Metasurface Mode Multiplexer

	LP_11b_	LP_01_	LP_11a_
input 1	–0.60	–43.1	–35.5
input 2	–43.8	–0.57	–34.8
input 3	–35.9	–33.6	–0.56

One might wonder whether the use of a resonant structure combined with nanoscale fabrication will result in significantly higher sensitivity to the misalignment of the fiber, making it difficult to assemble the system. Due to the reciprocal function of a MUX/DEMUX, the alignment tolerance is exactly the same as aligning the SMF or FMF fiber to another fiber in free space, and there are no additional drawbacks by having a highly integrated device. More details as In the measurement, we input light from found in the Supporting Information (Figure S2).

To test the scalability of the metasurface MUX to a higher number of modes, the adjoint analysis was repeated for six spatial modes (LP_01_, LP_11a_, LP_11b_, LP_02_, LP_21a_, and LP_21b_). The optimized device had a simulated insertion loss of ∼2.20 dB for all six modes at 1550 nm while retaining the same cavity size as the three-spatial mode MUX. There is a small increase in the lateral size of the phase profile (see Figure S3a), but there is no setback in terms of fabrication difficulty since it can still be easily written via electron beam lithography. A full insertion loss simulation over the C-band as well as crosstalk values at 1550 nm can be found in the Supporting Information (Figure S3b and Table S1, respectively).

### Experimental Characterization of the Metasurface Mode MUX

**Figure 3:**
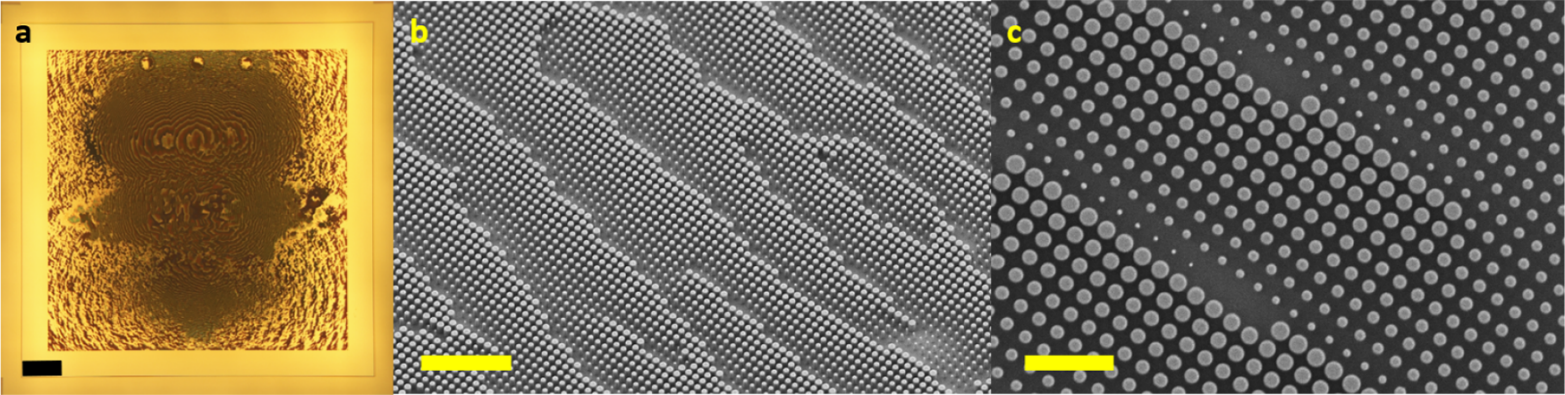
Images of the fabricated metasurface mode multiplexer. (a) Optical microscope image of the metasurface mode multiplexer (MUX) before the deposition of the Au mirror. Scale bar is 100 μm. (b) Angled and (c) bird’s-eye-view scanning electron microscope images of the mode MUX without the cladding and mirror layers at different zoom levels. Scale bar in (b) is 5 μm, and scale bar in (c) is 2 μm.

The six-mode metasurface MUX was fabricated according to the procedure in the Supporting Information (see Figure S4). The choice of materials and their physical parameters used in the fabrication are based on FDTD simulations (Ansys Lumerical Canada Ltd.). Consult the Methods section for a full list of parameters used to fabricate the MUX device. Due to having a large parameter space, the geometry of the nanopillars were chosen based on the nature of light propagation inside the metasurface. The amplitude and phase response of a nanopillar depends on the incident wavefront. In a cavity where the phase profile is imprinted on one of the mirrors, the incident light on any given nanopillar will not always be normal. To understand the angular sensitivity of the nanopillars, the phase shifts for different incident angles were simulated via FDTD in the Supporting Information (see Figure S5). This effect was generally negligible for cylindrical nanopillars with diameters under 400 nm and angles of incidence under 15^o^. In addition, by using polarization-insensitive nanostructures, our device performs the correct spatial mode conversion independent of the input polarization state. In practice, this allows both polarization states to be used as communication channels. Note, however, that this neglects any polarization dependence of the device as a whole. Measuring the extinction ratio of each mode can determine the polarization sensitivity of the MUX. The fabricated devices can be seen in Figure 3. The lateral size of the fabricated MUX is about 1 mm by 1 mm including the mirrors, which reiterates its compact form factor.

**Figure 4:**
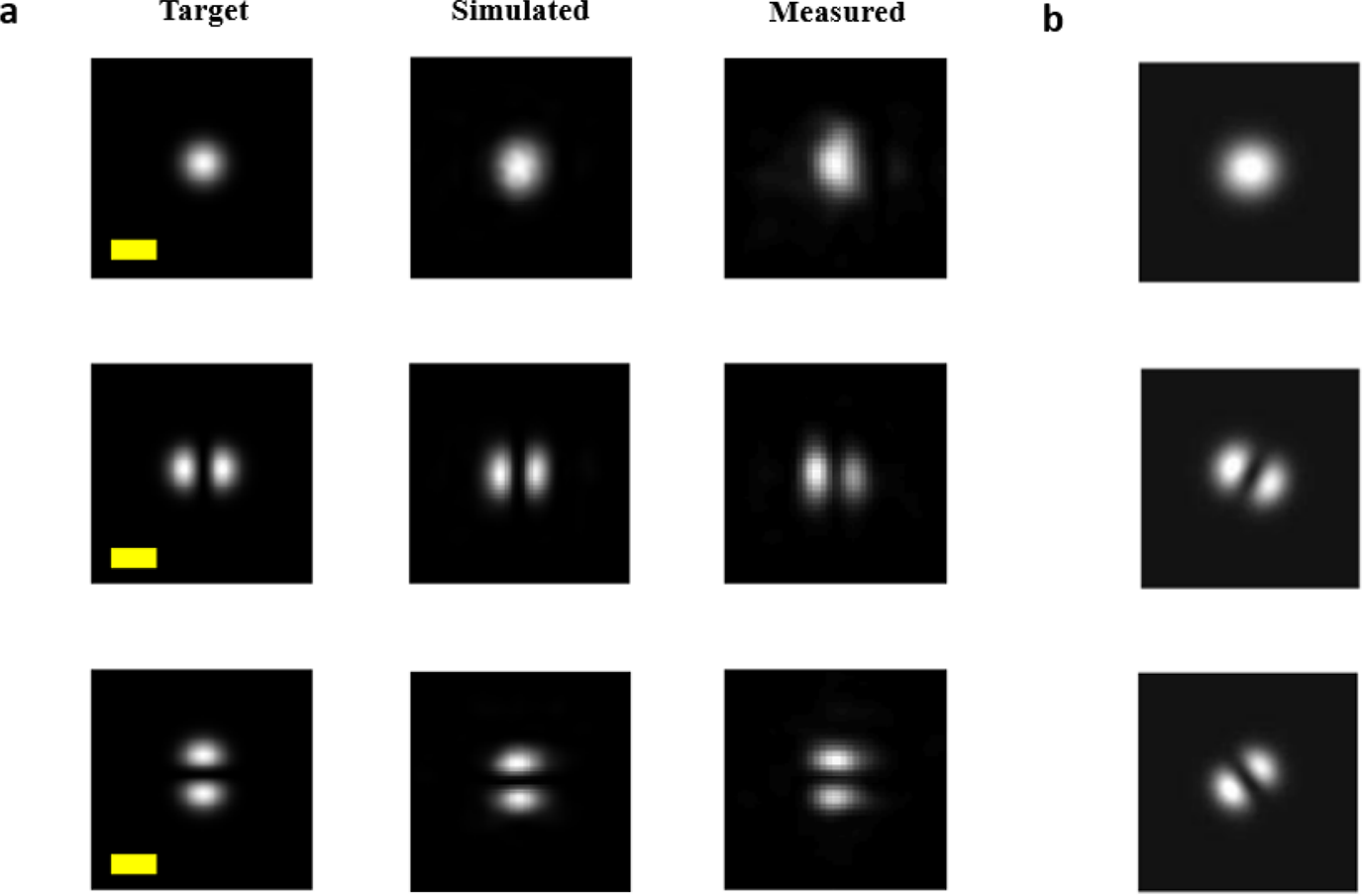
Characterization of converted modes of the metasurface mode multiplexer. (a) Mode profiles at 1550 nm. (Left) Intensity distribution of the ideal target linearly polarized modes of the few-mode fiber (FMF). (Middle) simulated and (right) measured output modes of the metasurface multiplexer. Camera images were interpolated twofold to improve resolution. Scale bars are all 10 μm and applies to all columns in (a). (b) Mode profiles at 1550 nm after coupling through the FMF.

We imaged the modes converted by the metasurface mode MUX to gauge their fidelity (refer to the Supporting Information for the measurement setup Figure S6). In the measurement, we input light from an external cavity laser into a SMF and aligned it to the input apertures of the MUX. Due to the design of the phase profile, the metasurface MUX can couple light directly from the input SMF fiber without any additional optics. An infrared objective and camera were used to capture the image of the converted modes coming out of the MUX. By exciting the leftmost, center, and rightmost apertures individually, we were able to observe the beam profiles corresponding to the LP_11b_, LP_01_, and LP_11a_ modes, respectively, at the output of the MUX. Figure 4a shows the ideal, calculated, and measured converted FMF modes from left to right. The mode images clearly show that our device can convert the inputs into target modes with reasonably clean shapes. The least pure mode seemed to be the LP_01_ mode. The distortion of the output modes and discrepancy between the measurement and the simulation results can be attributed to a multitude of reasons including fabrication errors, misalignment, and nanopillar loss mechanisms.^28^ In particular, we noticed that the cladding layer of the metasurface was not entirely flat. Undulations on the SU8 surface are imprinted onto the gold mirror upon deposition and thus introduce distortions to the intended phase profile. Despite this, the mode shapes from the mode MUX were clean and, as a result, we were able to excite the desired modes in the FMF with high purity as seen in Figure 4b. Due to the circular symmetry of the FMF fiber, there is mode coupling between LP_11a_ and LP_11b_ modes within the fiber; thus, the orientation of the two lobes at the output was not aligned with the orientation of the lobes at the input. However, there was very little mixing between LP_01_ and the two LP_11a,b_ modes, which is further verified below.

**Figure 5:**
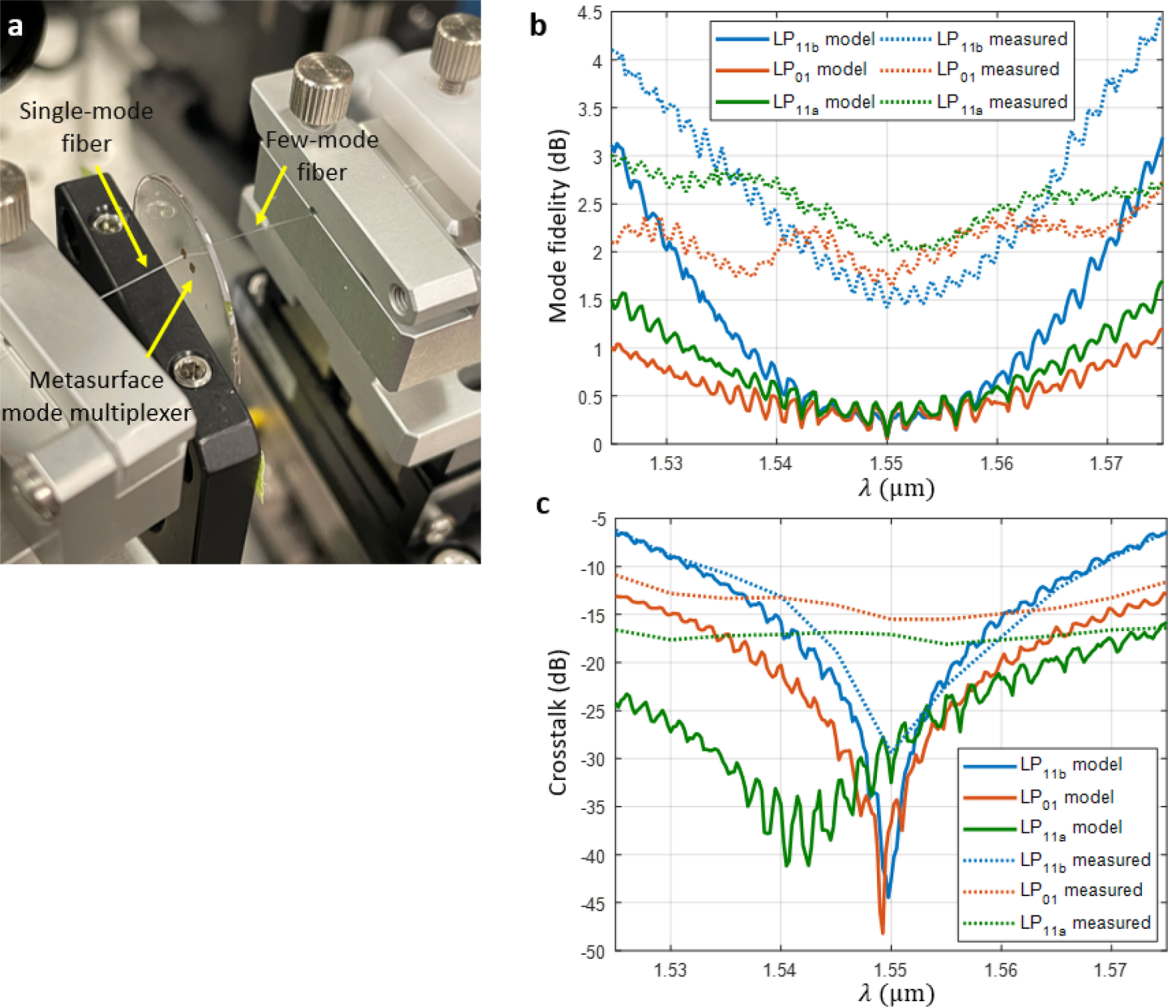
Mode fidelity and crosstalk performance of the metasurface mode multiplexer. (a) Digital camera image of the metasurface mode multiplexer coupled to a single-mode fiber and few-mode fiber. There are two metasurface multiplexers on the substrate (one indicated by arrow). (b) Simulated and measured mode fidelity of the modes from the metasurface multiplexer. (c) Simulated and measured crosstalk of the metasurface multiplexer. Crosstalk is defined as the power in the incorrect mode over the power in the correct mode.

To quantify the purity of these modes (i.e., mode fidelity), we devised a method that measures the mode insertion loss to the FMF and internal device loss. The difference of the two losses yields the mode fidelity. The device was coupled to a SMF on one side and a FMF on the other as shown in Figure 5a. The detailed measurement setup is explained in the Supporting Information (Figure S7). The insertion loss was measured first by coupling the light coming out of the metasurface MUX to a 10 m long FMF and aligned to optimize for the mode shape and output power. This length is enough to strip out any leaky higher-order mode that might be undesirably excited. This is evident by the pure modes shown in Figure 4b. The measured insertion losses for each mode at 1550 nm are: LP_01_ = 12.2 dB, LP_11a_ = 12.4 dB, and LP_11b_ = 12.4 dB. Second, to measure the internal device loss, the FMF was removed, and instead, a small pin-hole aperture was placed close to the device output aperture. This ensures that only light from the device output aperture is measured. Thus, internal device loss is defined as all loss that comes from within the device itself. The measured internal device losses at 1550 nm are: LP_01_ = 10.5 dB, LP_11a_ 10.2 = dB, and LP_11b_ 10.9 = dB. To obtain the mode fidelity, the internal device loss is subtracted from the insertion loss. The mode fidelity values of the metasurface MUX are shown in Figure 5b measured over the C-band overlaid on simulated values. At 1550 nm, the measured values are: LP_01_ = 1.77 dB (67%), LP_11a_ = 2.18 dB (61%), and LP_11b_ = 1.42 dB (72%). These results are within 2 dB of the simulated values, which represent reasonably good agreement with the purity of the profiles at the FMF output. Note that the small ripples in the insertion loss curves are repeatable and do not change with the length of the FMF as they arise from the free spectral range of the metasurface cavity. Although the measured insertion loss, which is the original figure of merit from the adjoint analysis, is significantly higher than the simulated values, it is critical to distinguish that mode fidelity is the key parameter to determine the capability of a metasurface to precisely transform an input mode into the desired shape. Internal device loss includes Fresnel and diffractive losses as well as fabrication imperfections. The quality of the fabricated device has room for improvement, and as a result, so does the overall insertion loss. Because the mode fidelity of our device is good, the measured crosstalk values also remain low. The crosstalk values of the metasurface MUX at 1550 nm are: LP_01_ = −15.5 dB, LP_11a_ = −17.1 dB, and LP_11b_ = −29.3 dB. This measurement was based on the ratio of power in the undesired mode over the power in the target mode in the FMF^29^ (see the Supporting Information). Note that this is slightly different from the definition in Table 1, where power was normalized to the input. In addition, with this method, we do not distinguish the crosstalk between LP_11a_ and LP_11b_ modes since they easily couple in the FMF, and due to this reason, the values are different from Table 1. The LP11b crosstalk is more sensitive to the wavelength than other modes because of mode symmetry and its orientation. In our design, the incident beam comes at an angle of 12^o^ to the input aperture and, after interaction with the metasurface, comes out of the output aperture at 0^o^. Due to the dispersive nature of this metasurface, as the incident light changes wavelength, the position of the output beam will shift slightly along the direction of the initial beam tilt. Thus, certain LP modes (the LP_11b_) will be more affected by this shift as the wavelength changes versus the other modes. The full crosstalk data over the C-band can be found in Figure 5c overlaid on top of the simulated values. Although our device was optimized at 1550 nm, its performance within the bandwidth of 1540–1560 nm is relatively stable. For all modes, the mode fidelity varies less than 1 dB, and the crosstalk stays below 15 dB for most of the bandwidth. This range falls within dense WDM systems, which indicates that our device does have WDM compatibility.

The polarization sensitivity of this device was also tested. The laser source was connected to a fiber-based polarization controller before being coupled into the metasurface MUX (details of the setup can be found in the Supporting Information Figure S8). Insertion losses were measured for both polarizations, and we found no significant deviation due to the polarization state. Mode images coming out of the FMF also confirmed this observation. Moreover, we measured the output extinction ratio of each mode at 1550 nm and found that it ranged from 24–27 dB for all modes (refer to the Supporting Information Figure S9). This is defined as *r*_e_ = *P*_2_/*P*_1_, where *P*_2_ is the optical power of the desired polarization and *P*_1_ is the optical power of the undesired polarization. This implies that the MUX design is reasonably polarization-insensitive.

## Conclusions

We have introduced a new kind of metasurface mode MUX based on a cavity architecture. In mode multiplexing, where the rich interaction of light is required to shape the wavefront, the cavity achieves this while also enabling resonant effects and a fabrication process that does not require complicated alignment of the phase pattern. The adjoint analysis of the metasurface phase profile is an efficient way to optimize the phase while intuitively addressing other design requirements such as mode-dependent loss. Compared to current mode multiplexing schemes, our metasurface MUX is scalable, easy to fabricate due to single-step lithography, and highly compact with a size of about 1 mm^2^ for a six-mode (three-spatial mode) MUX. Doubling the number of multiplexed modes is possible while keeping the cavity size of the metasurface the same and slightly increasing the lateral dimensions of the phase profile. This has nearly no drawbacks in the fabrication difficulty unlike alternative multiplexing techniques. Based on this platform, our fabricated metasurface MUX can convert input SMF modes into FMF modes with good fidelity of up to 72%. Although the total insertion loss is higher than alternative MUX designs, there is much room for optimization in the fabrication, nanopillar design, and cladding layer. In addition, depending on material availability, the cladding layer can be made thinner to improve the diffraction efficiency of the nanopillars. Metasurfaces offer a unique way to increase the capacity of today’s communication using an ultracompact device. They can also be topologically optimized to be birefringent and have control over dispersion, which could open new avenues for MUX devices to address the ever-growing need for higher data rates.

## Methods

### Adjoint Analysis Simulation

Light propagation was modeled assuming a scalar field using the Rayleigh–Sommerfeld diffraction formulation^24^ and calculated efficiently using discrete Fourier transforms.^25^ The design parameters of the MUX are based on optimizing the device at 1550 nm and other physical limitations. The thickness of the substrate *d* was set to 525 μm to account for available fused silica substrate sizes. The aperture diameters for the top *T*_0_ and bottom *T*_1_ reflectors were chosen based on the mode diameters of the input and output modes. To minimize clipping of the input and output beams while retaining effective mirror surfaces, the diameters for the top and bottom apertures were 30 and 50 μm, respectively. The input and output fiber were designed to be 45 μm away from the surface of the device to allow space for alignment. The discretization of the phase masks and electric fields in the simulations was set to 1 μm to provide a balance between high spatial resolution and computational speed. After the phase masks were optimized, they were interpolated to a grid with 500 nm spacing, which corresponds to the unit cell size of the nanopillars used to fabricate the MUX.

### Fabrication Parameters

In reference to Figure 1b, the mirror layer was made of 250 nm thick gold (both sides of substrate), the dielectric was chosen to be 575 nm thick amorphous silicon (a-Si) due to its high refractive index and low loss at 1550 nm, the cladding was chosen to be 1.9 μm thick SU8 due to its high transparency, the substrate was 525 μm thick fused silica, and the unit cell of each nanopillar was 500 nm.

### Mode Fidelity Calculation

The measured mode fidelity in Figure 5b was the result of subtracting the measured internal device loss from the measured insertion loss. The insertion loss is the power that is coupled into the desired FMF mode relative to the amount of power launched from the SMF input. It can be broken down into two losses: (1) the amount of power lost after exiting the device at the output aperture and (2) the amount of power at the FMF lost due to mode shape mismatch. The former is defined as internal device loss, and the latter is known as mode fidelity. Note that mode fidelity also includes any misalignment between the MUX device and the output fiber, such as offset, tilt, and focus.

### Characteristics of the Few-Mode Fiber

The FMF used in this work has a core radius of 10.2 μm and an index delta of 0.52%. Calculations of the fiber modes show that the LP_01_ and LP_11_ mode groups are supported by the fiber. The mode profiles are shown in Figure 4a (left column). Note that these are also the target modes used in the adjoint analysis optimization.

## Supporting Information Available

Derivation of the gradient of the figure of merit for adjoint analysis; alignment tolerance of fiber to the metasurface mode multiplexer (MUX); scalability of the metasurface mode MUX; fabrication procedure for metasurface mode MUX; finite-difference time domain (FDTD) library of a-Si nanopillars at λ = 1550 nm; measurement setup for imaging mode conversion; measurement setup for characterizing device loss; polarization dependence measurement setup; polarization dependence of the metasurface MUX; crosstalk measurement setup (PDF)
