# Supplementary material for: Adjoint-optimized metasurfaces for compact mode-division multiplexing
Source: ACS Photonics. 2022 Mar 7;9(3):929–37. doi: 10.1021/acsphotonics.1c01744 (PMC8931746; doi:10.1021/acsphotonics.1c01744)
Supplement: Supplementary file 1 — ph1c01744_si_001.pdf [file ph1c01744_si_001.pdf]

Adjoint optimized metasurfaces for compact mode-division multiplexing: Supporting Information

Jaewon Oh[1], Kangmei Li[2], Jun Yang[2], Wei Ting Chen[1], Ming-Jun Li[2], Paulo Dainese[2] and Federico Capasso[1]

[1]Harvard John A. Paulson School of Engineering and Applied Sciences, Harvard University, Cambridge, Massachusetts 02138, USA

[2]Corning Inc., Painted Post, New York 14870, USA

Pages S1-S12
Figures S1-10

1. Derivation of the gradient of the figure of merit for adjoint analysis

The following is derived assuming the non-resonant physical model for better physical intuition. The final gradient in equation (S9) still applies for the cavity model except the superscript (i) is dropped since the derivative is no longer calculated at each plane due to the steady state field assumption in the device.

Here we consider a stack of N equidistantly spaced transmission masks $T^{(i)} = \exp\left(j\phi^{(i)}\right), i = 1 \dots N$ where $\phi^{(i)}$ is the ith mask. The field at different position, illustrated as the dashed lines, are $u^{(i)}, i = 0 \dots N+1$. Then $u^{(0)}$ corresponds to the input field, and $u^{(N+1)}$ corresponds to the output field. U is the free space propagator.

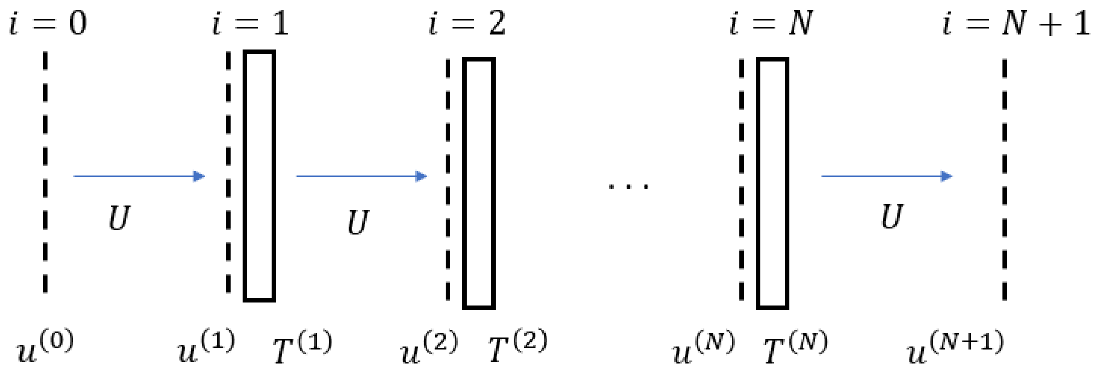

Fig. S1 Schematic of light propagation within a series of phase plates. At a given instance along propagation, the field is denoted by a vertical dashed line. Phase plates are denoted by vertical rectangles.

Calculating the field under the scalar field diffraction is

$$\begin{aligned} u^{(1)} &= Uu^{(0)} \\ u^{(2)} &= UT^{(1)}u^{(1)} \\ u^{(i)} &= UT^{(i-1)}u^{(i-1)} \\ u^{(N+1)} &= UT^{(N)}u^{(N)} \end{aligned} \tag{S1}$$

Our figure of merit is the overlap integral of the field after the last plane:

$$f(u) = \left|\langle u^{tar} | u^{(N+1)} \rangle\right|^2 = \left|u^{tar\dagger} u^{(N+1)}\right|^2 \tag{S2}$$

where u^{tar} is the desired target field located at plane $i = N + 1$

We can rewrite Equation (S1) as a large linear equation

$$A(\phi)u = b \tag{S3}$$

where

$$A(\phi)=\begin{pmatrix}1 & 0 & 0 & \cdots & 0\\ -UT^{(1)} & 1 & 0 & \cdots & 0\\ 0 & -UT^{(i-1)} & 1 & \cdots & 0\\ \vdots & \vdots & \vdots & \ddots & \vdots\\ 0 & 0 & \cdots & -U^{(N+1)}T^{(N)} & 1\end{pmatrix}, u=\begin{pmatrix}u^{(1)}\\ u^{(2)}\\ u^{(i)}\\ \vdots\\ u^{(N+1)}\end{pmatrix}, b=\begin{pmatrix}Uu^{(0)}\\ 0\\ 0\\ \vdots\\ 0\end{pmatrix}$$

Our goal is then to calculate $\frac{\partial f}{\partial \phi^{(i)}}$ given that f is an explicit function of u, and u satisfies the linear equation (S3).

From adjoint analysis [1], the derivative of the figure of merit with respect to the design variable can be expressed as:

$$\frac{df}{d\phi^{(i)}} = -2\text{Re}\left\{\frac{\partial f}{\partial u}\frac{du}{d\phi^{(i)}}\right\} = -2\text{Re}\left\{\left(A^{-\dagger}\left(\frac{\partial f}{\partial u}\right)^{\dagger}\right)^{\dagger}\left(\frac{\partial A}{\partial \phi^{(i)}}\right)u\right\} \tag{S4}$$

To take a closer look at each term

$$\left(\frac{\partial f}{\partial u}\right)_i = \delta_{i,N+1}\left(u^{tar\dagger}u^{(N+1)}\right)^{*}u^{tar\dagger}$$

$$\left(\frac{\partial A}{\partial \phi^{(i)}}\right)_{jk} = -U\frac{dT^{(i)}}{dz^{(i)}}\delta_{j,i+1}\delta_{k,i}$$

Plugging these partial derivatives back, Eqn. (S4) becomes

$$\frac{\partial f}{\partial \phi^{(i)}} = 2\text{Re}\left\{\left(u^{tar\dagger}u^{(N+1)}\right)^{*}\left(A^{-\dagger}u^{tar}\right)^{\dagger(i+1)}U\frac{dT^{(i)}}{d\phi^{(i)}}u^{(i)}\right\} \tag{S5}$$

Now we define the adjoint field $v \equiv A^{-\dagger}u^{tar}$ which is physically the field backpropagating from the target field. Clearly at $i = L+1$, $v^{(L+1)} = u^{tar}$.

For some plane i, $v^{(i)} = T^{(i)\dagger}U^{\dagger}v^{(i+1)}$ which is equivalent in saying the adjoint field at some plane is equal to the field after it in space that has been backpropagated and multiplied by the conjugate of the transmission mask. Rearranging yields $v^{(i)\dagger}T^{(i)\dagger} = v^{(i+1)\dagger}U$ which can be subbed in (S5)

$$\frac{\partial f}{\partial \phi^{(i)}} = 2\text{Re}\left\{\left(u^{tar\dagger}u^{(N+1)}\right)^{*}v^{(i)\dagger}T^{(i)\dagger}\frac{dT^{(i)}}{d\phi^{(i)}}u^{(i)}\right\} \tag{S6}$$

Since $T^{(i)} = \exp\left(j\phi^{(i)}\right)$ then $\frac{dT^{(i)}}{d\phi^{(i)}} = jT^{(i)}$ or $T^{(i)\dagger}\frac{dT^{(i)}}{d\phi^{(i)}} = j$ such that (S6) simplifies to

$$\frac{\partial f}{\partial \phi^{(i)}} = -2\,\mathrm{Im}\left\{\left(u^{tar\dagger} u^{(N+1)}\right)^* v^{(i)\dagger} u^{(i)}\right\} \tag{S7}$$

Notice that $\left(u^{tar\dagger} u^{(N+1)}\right)^*$ is just a complex number and serves the role to correct the phase. To paraphrase Eqn. (S7), the dependence of the objective function f on the phase value on the ith phase plate $\phi^{(i)}$ depends only on the multiplication of the forward field $u^{(i)}$ and the adjoint field $v^{(i)}$ at the same location.

If we have multiple objectives, then we can create an overall objective based on the sub-objectives $f = f(f_1, f_2, \dots, f_k)$. For example, the simplest objective function can be just the average of all objectives. Then

$$f = \frac{1}{K}\sum_{k=1}^{K} f_k \tag{S8}$$

Then using chain rule, we have

$$\frac{\partial f}{\partial \phi^{(i)}} = \frac{1}{K}\sum_{k=1}^{K}\frac{\partial f_k}{\partial \phi^{(i)}} = -\frac{2}{K}\sum_k \mathrm{Im}\left\{\left(u_{(k)}^{tar\dagger} u_{(k)}^{(N+1)}\right)^* v_{(k)}^{(i)\dagger} u_{(k)}^{(i)}\right\} \tag{S9}$$

Similar results can be derived for different objectives, such as $f = \max_k f_k$ if we want to minimize the worst-case loss.

2. Alignment tolerance of fiber to the metasurface mode multiplexer (MUX)

Here we would like to quantify the alignment tolerance of the input and output fibers with respect to the metasurface device. Since our device is a mode conversion device, the additional coupling loss to the few-mode fiber (FMF) due to its misalignment can be simply calculated as the insertion loss between the ideal FMF mode and the actual FMF mode outputted by the device because they will be misaligned by the same amount. Since a mode conversion device is reciprocal in function (either with single-mode fiber (SMF) as input and FMF as output, or vice versa), the alignment tolerance on the SMF side is the same. To further verify this, we calculate the insertion loss when the input SMF is laterally offset by some amount. Here we take the LP_{11b} mode as an example. The result is shown below on the left (Fig. S2a). The blue and orange lines correspond to cases where the input SMF fiber is laterally offset in the x or y direction, respectively, and the insertion loss to the LP_{11b} mode is simulated for the fixed output FMF. The green dashed line corresponds to directly calculating the insertion loss between two SMFs that are offset by the same amount. One can see that three curves are essentially equivalent. The same argument

applies to the tilt tolerance (Fig. S2b). Modeling results are shown below on the right. Thus, even though our device is highly integrated, there are no additional drawbacks in alignment for the input and output fiber.

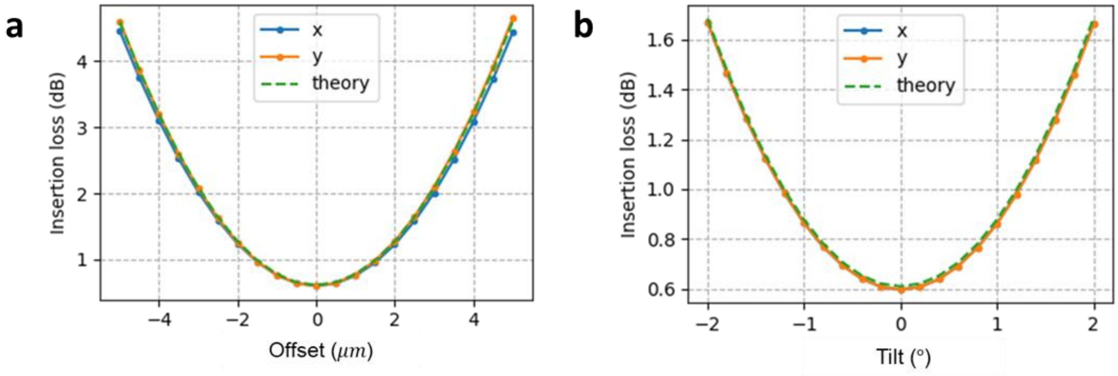

Fig. S2 Alignment tolerance calculations of the metasurface mode MUX for **a** lateral offsets of the SMF and **b** for angular tilts of the SMF.

3. Scalability of the metasurface mode MUX

An advantage of the cavity metasurface MUX design over existing MUX technologies is the ability to scale to a greater number of modes without significant reduction in performance and none in fabrication effort. To demonstrate this, we designed a 12-mode (6 spatial modes and 2 polarization states) MUX using the adjoint analysis technique described in the main text. Figure S3a and b show the optimized phase profile and simulated insertion loss over wavelength, respectively. Here the non-resonant design is shown due to having better bandwidth performance than a resonant design. Table S1 shows the crosstalk matrix at 1550 nm. Note that, despite scaling to twice as many modes of the fabricated design, the substrate thickness (i.e. cavity size) remains the same and the lateral size increases by an amount that can still be easily written by electron beam lithography.

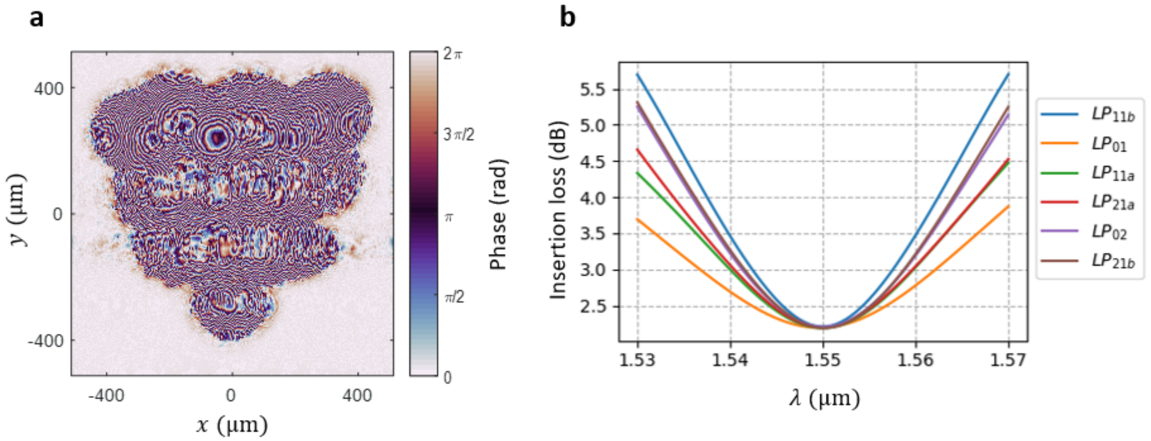

Fig. S3 Adjoint analysis of a 12-mode metasurface mode MUX. a The optimized phase profile **b** The simulated insertion loss across the C-band.

Table S1. Simulated insertion loss and crosstalk (dB) at 1550 nm of the 12-mode metasurface mode MUX

	LP_{11b}	LP_{01}	LP_{11a}	LP_{21a}	LP_{02}	LP_{21b}
Input 1	-2.21	-34.7	-40.8	-43.9	-35.4	-33.9
Input 2	-52.3	-2.19	-41.0	-24.2	-16.6	-37.5
Input 3	-49.7	-36.8	-2.19	-34.9	-47.0	-34.3
Input 4	-39.2	-22.2	-28.9	-2.19	-25.7	-48.2
Input 5	-39.0	-21.8	-42.2	-20.7	-2.20	-42.6
Input 6	-34.3	-41.5	-37.8	-46.4	-40.9	-2.19

4. Fabrication procedure for metasurface mode MUX

Refer to Fig. S4. First a fused silica substrate of thickness 525 μm was cleaned via acetone and isopropyl alcohol (IPA) wash. Amorphous silicon (a-Si) was deposited using plasma-enhanced chemical vapor deposition (PECVD) to a thickness of 575 nm. Then hydrogen silsesquioxane (HSQ) was spin coated over the wafer as e-beam resist. The MUX were patterned using electron beam lithography and was developed in 25% tetramethylammonium hydroxide (TMAH) for 25 seconds. A small thickness of HSQ is left on top of the MUX patterns that served as the etch mask for reactive ion etching of the a-Si pillars. After the pillars were etched, the remaining HSQ thickness was close to negligible. The form the cladding layer, SU8 polymer was spin coated on top of the pillars at a speed to yield 1.9 μm thickness (relative to the substrate). The SU8 was UV-cured over a prolonged exposure time. Then the apertures for the front side of the device was pattern via photolithography with S1818 resist. A 250 nm-thick gold layer was deposited using e-beam evaporation. Finally, lift-off formed the apertures. Aperture patterning was then repeated for the backside.

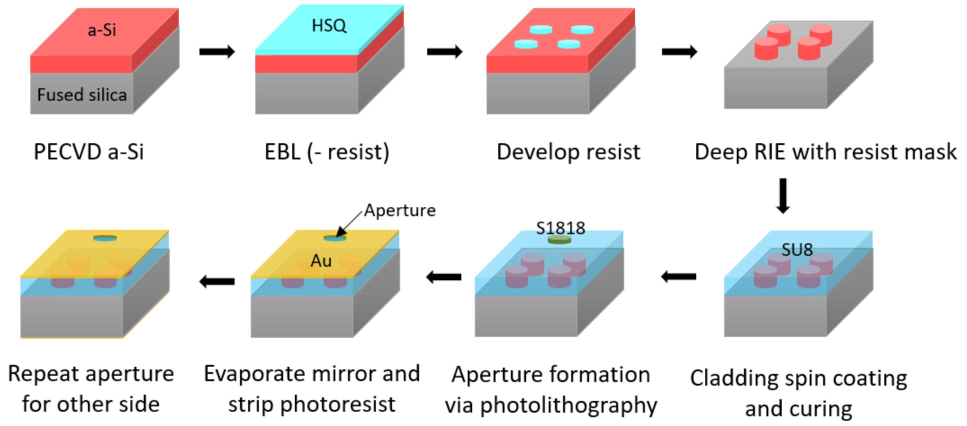

Fig. S4 Fabrication steps for the metasurface mode MUX

5. Finite-difference time domain (FDTD) library of a-Si nanopillars at $\lambda = 1550$ nm

FDTD simulations (Ansys Lumerical Canada Ltd.) of the nanopillar library were conducted assuming Bloch boundary conditions with angles of incidence ranging from 0° to 15°. Note that this does not necessarily guarantee angle insensitivity. When combined to make a certain phase profile, the nanopillars as a whole can have an angle-dependent response (structural birefringence) that cannot be predicted from this simulation alone since the Bloch boundary conditions are no longer accurate. However, for low spatial frequency phase patterns the approximation is valid. Figure S5 shows that for angles below 15°, the phase shift response is insensitive.

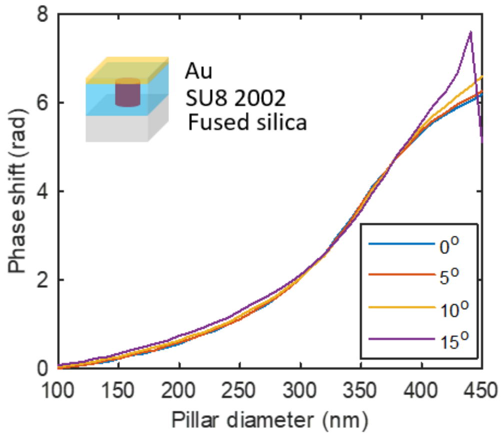

Fig. S5 FDTD analysis of the angular sensitivity of dielectric a-Si nanopillars. The angle of incidence is noted in the legend.

6. Measurement setup for imaging mode conversion

Measurement setup for imaging the modes coming out of the metasurface MUX (Fig. S6). Incident light with wavelength 1550 nm from the tunable laser was fiber coupled to a single mode fiber (SMF) and precisely aligned to the input ports of the MUX. Depending on which input port was excited, a unique LP mode was emitted from the output aperture of the MUX. The image of the mode was captured by the InGaAs camera.

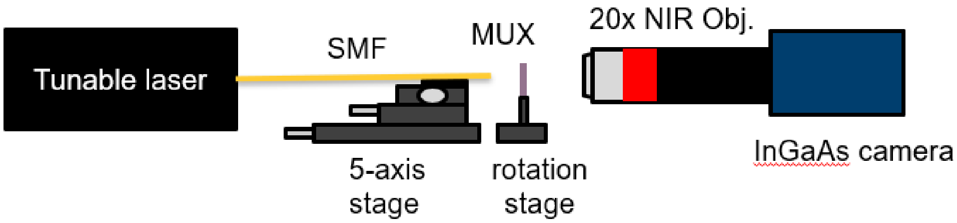

Fig. S6 Setup for imaging converted modes from the metasurface mode MUX.

7. Measurement setup for characterizing device loss

In the first setup (Fig. S7a), the output mode from the MUX is coupled to a corresponding FMF with matching mode distribution and the coupled power is measured at the end of the FMF with a detector. This is done for each input just as in S6. The power captured from this output represents purely the light in the target mode assuming the loss in the FMF is negligible. This is defined as insertion loss in the main text. In the second setup (Fig. S7b), the total optical power coming from the output port of the MUX is measured. This is done for each input port by adding a pinhole that only allows light from the output aperture of the MUX to be captured (light exiting the boundaries of the MUX mirrors is blocked). The power captured from this output represents loss from within the device including unwanted diffraction, material absorption, as well as fabrication imperfections. This is defined as internal device loss in the main text.

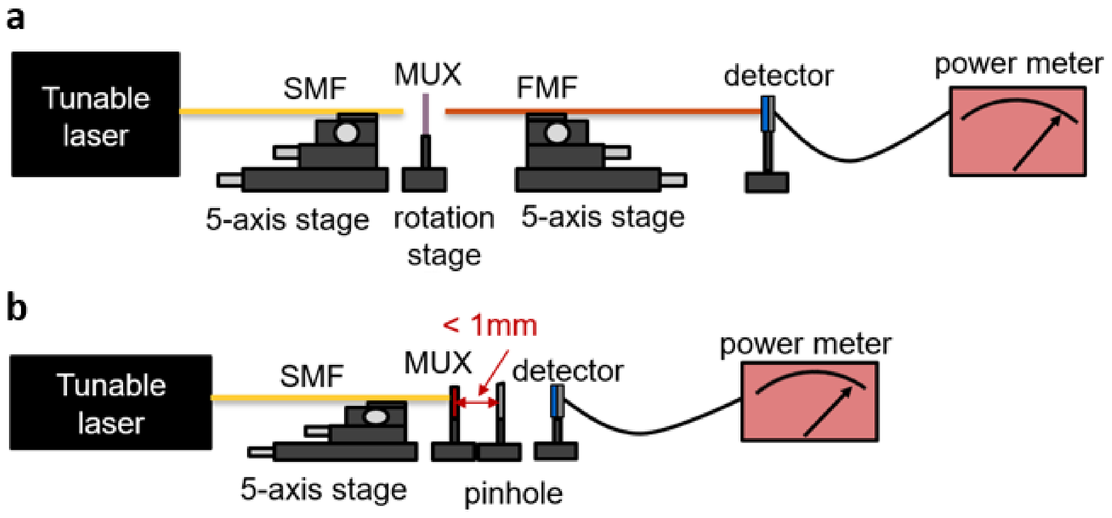

Fig. S7 Two measurement setups used to measure mode fidelity of the metasurface MUX. a Setup used to measure insertion loss. The FMF is 10 m long. **b** Setup used to measure internal device loss. The difference of the two yields the mode fidelity.

In order to estimate the mode fidelity of the MUX, one can subtract the power difference measured from the first setup and the second setup. Finding this number for each mode represents the fidelity of the mode profiles converted by the MUX. This is true since in Fig. S7b we capture all the loss from within the device itself and in Fig. S7a we capture the total loss from SMF input to the end of the FMF fiber. The loss while propagating the FMF

is negligible thus the difference between the two losses must come from the mode overlap of the converted mode by the MUX and the desired target mode of the FMF.

8. Polarization dependence measurement setup

The setup shown below (Fig. S8) was used to measure the extinction ratio of each mode of the metasurface mode MUX. This was done to gauge the polarization dependence of the device. In this setup there is an electric polarization controller that controls the source polarization incident on the metasurface MUX and there is a polarizer at the output side that analyzes the polarization state. For a given input polarization and mode, the power is measured when the second polarizer is co-polarized and then the power is measured again when the second polarizer is cross-polarized for the same mode.

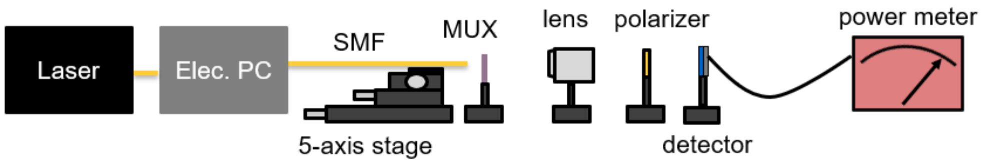

Fig. S8 Setup for determining the polarization dependence of the metasurface mode MUX.

9. Polarization dependence of the metasurface MUX

In order to gauge the polarization dependence of our metasurface mode MUX, the extinction ratio was measured for each mode and then FMF mode images were captured for different polarizations. The extinction ratio is defined as the power of mode with the desired polarization over the power of the mode in the unwanted polarization. Results were obtained from the setup in Fig. S8. For the FMF mode images, the polarizer and detector at the output side is replaced by the FMF and a camera in Fig. S8.

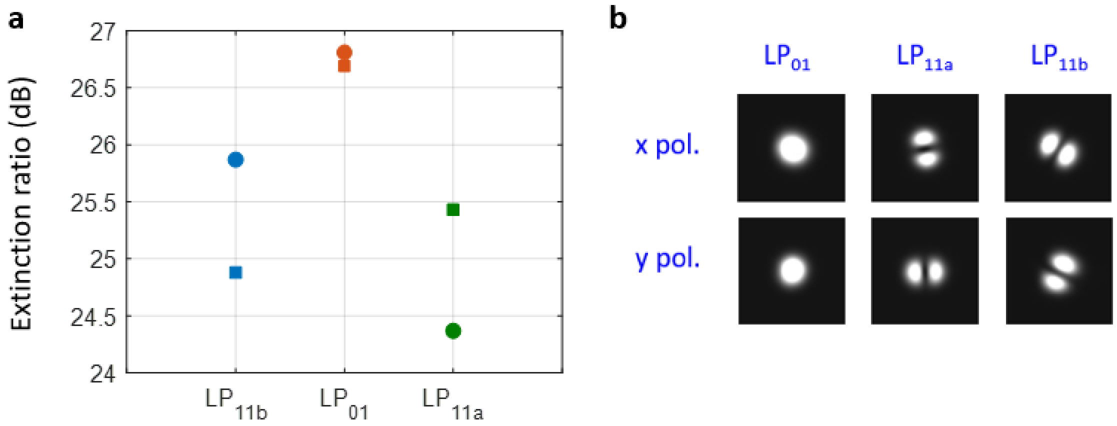

Fig. S9 Polarization dependence of the metasurface mode MUX for linear polarization. **a** Extinction ratio measurement of each converted mode. Circles indicate the desired polarization is x-polarized and squares indicate the desired polarization is y-polarized. **b** Mode images from the FMF after launching linear polarizations from the metasurface MUX

10. Crosstalk measurement setup

A schematic of the measurement setup used for the crosstalk of the metasurface mode MUX is presented below in Fig. S10. This method is based on Ref. 29 in the main text. A vector network analyzer (VNA) is used to control the modulation frequency of a narrow linewidth continuous-wave source via an intensity modulator (IM). The modulated light is launched into the metasurface mode MUX and the output modes are then coupled to the fiber under test (FUT) i.e. the FMF. At the end of the FMF, a photodetector (PD) is used to convert the optical signal back to an electrical signal. This is the transmission over the range of modulation frequency. Due to crosstalk of the MUX device, the light before the FMF will be a mix of the two LP mode groups. After this mix is launched into the FMF, the input pulse will separate in time based on modal dispersion. Because a narrow linewidth source is used, chromatic dispersion can be neglected and a Fourier transform of the output pulse will decompose the signal into its modal constituents weighted by the relative power in each mode. Based on the definition of crosstalk given in the main text, the crosstalk can be calculated from these weights. Note that this method only distinguishes between the LP_{01} and LP_{11} mode groups.

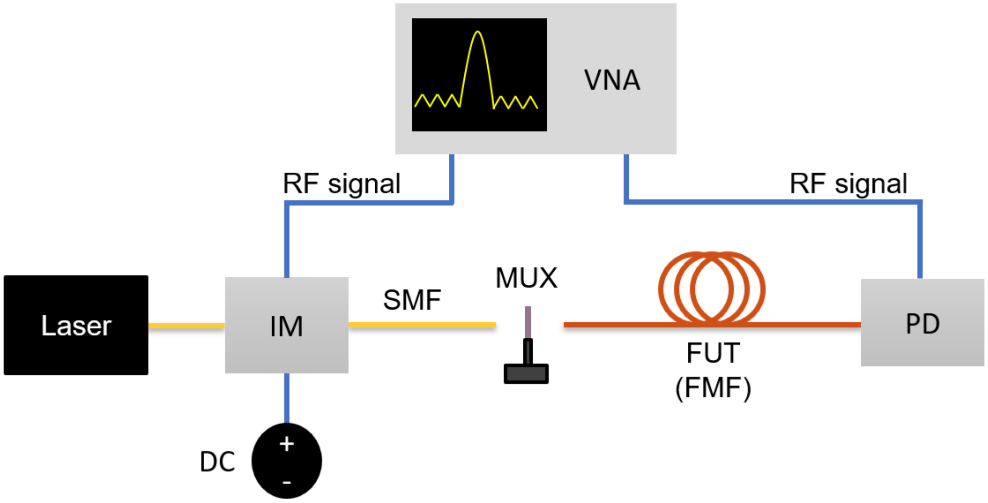

Fig. S10 Setup for determining the crosstalk performance of the metasurface mode MUX

References

1. A. Piggott, “Automated design of photonic devices,” PhD thesis (Department of Electrical Engineering, Stanford University, 2018).
